# A Music Playback Algorithm Based on Residual-Inception Blocks for Music Emotion Classification and Physiological Information

**DOI:** 10.3390/s22030777

**Published:** 2022-01-20

**Authors:** Yi-Jr Liao, Wei-Chun Wang, Shanq-Jang Ruan, Yu-Hao Lee, Shih-Ching Chen

**Affiliations:** 1 Department of Electronic and Computer Engineering, National Taiwan University of Science and Technology, Taipei 106, Taiwan; m10902107@mail.ntust.edu.tw (Y.-J.L.); sjruan@mail.ntust.edu.tw (S.-J.R.); 2 Department of Humanities and Social Sciences, National Taiwan University of Science and Technology, Taipei 106, Taiwan; vgnwang@mail.ntust.edu.tw; 3 Department of Physical Medicine and Rehabilitation, Shuang Ho Hospital, Taipei Medical University, Taipei 106, Taiwan; 15411@s.tmu.edu.tw; 4 Department of Physical Medicine and Rehabilitation, Taipei Medical University Hospital, Taipei 106, Taiwan; 5 School of Medicine, College of Medicine, Taipei Medical University, Taipei 106, Taiwan

**Keywords:** convolutional neural networks, emotion classification, deep learning, music selection module, physiological data

## Abstract

Music can generate a positive effect in runners’ performance and motivation. However, the practical implementation of music intervention during exercise is mostly absent from the literature. Therefore, this paper designs a playback sequence system for joggers by considering music emotion and physiological signals. This playback sequence is implemented by a music selection module that combines artificial intelligence techniques with physiological data and emotional music. In order to make the system operate for a long time, this paper improves the model and selection music module to achieve lower energy consumption. The proposed model obtains fewer FLOPs and parameters by using logarithm scaled Mel-spectrogram as input features. The accuracy, computational complexity, trainable parameters, and inference time are evaluated on the Bi-modal, 4Q emotion, and Soundtrack datasets. The experimental results show that the proposed model is better than that of Sarkar et al. and achieves competitive performance on Bi-modal (84.91%), 4Q emotion (92.04%), and Soundtrack (87.24%) datasets. More specifically, the proposed model reduces the computational complexity and inference time while maintaining the classification accuracy, compared to other models. Moreover, the size of the proposed model for network training is small, which can be applied to mobiles and other devices with limited computing resources. This study designed the overall playback sequence system by considering the relationship between music emotion and physiological situation during exercise. The playback sequence system can be adopted directly during exercise to improve users’ exercise efficiency.

## 1. Introduction

Recent years have seen the burgeoning development of measurement technology and the application of this technology to wearable devices. With the increasing demand for physical exercise, functionality requirements for wearable devices have become more critical. Even though there are many studies attesting to the positive effects of exercise [1,2,3,4], most people still ignore the importance of physical exercise. The fact that hypomotility has become the fourth risk factor for global death was indicated by the World Health Organization (WHO). Approximately 60–85% of adults live statically worldwide, and two-thirds of children lack exercise. This situation increases the risk of cardiovascular disease, diabetes, and obesity compared to those who exercise regularly. Additionally, it leads to over two million people deaths every year. From the perspective of health, people who have good cardio respiratory endurance can exercise longer, do not get tired easily, and avoid disease. The central purpose of this study is to extend people’s sports time by exposure to suitable music.

The concept that people who listen to different types of music will change emotions and physical states has been widely accepted. Several studies have shown the relationship between psychology and physiology [5,6]. According to Bason et al. [7], the heart rate changes mainly for the following reasons. First, the heart rate is changed by the external auditory stimulation that leads to the neuron coupling into the cardiac centers of the brain, further arousing the sinus entrainment of rhythms. Another cause of changing the heart rate is the autonomic nervous system (ANS) that controls and sustains homeostasis in our body, such as blood pressure, body temperature, and sleep qualities. It mainly consists of the parasympathetic nervous system (PNS) and sympathetic nervous system (SNS). Additionally, it is typically distinguished by opposing characteristics. For instance, in an emergency state, the SNS increases the heart rate, but on the other hand, the PMS typically retards the heart rate in the static state. Some studies indicate that quality of life can be improved by differenttypes of music, such as raising sleep quality, relieving pressure, supporting exercise, and enhancing brain liveliness [8,9,10]. In summary, we could further infer that there supposedly is a connection between music stimuli and heart rate.

According to the above-mentioned factors, listening to different music genres changes one’s emotions and heart rate, which is a kind of music therapy method. There are some benefits of music therapy, such as socialization, cognition, emotion, and neuron motor function [11]. Continuing music therapy research has led to many new and fascinating applications in sports and autistic and handicapped fields. According to Van Dyck E et al. [12], music rhythm can affect running cadence. In other words, the slower rhythm of music brings out a decrease in running cadence; on the other hand, the faster rhythm of music gives rise to an increase in running cadence. Moreover, another significant research of promoting exercise efficiency is proven by Karow et al. [13]. They provided extensive discussions of the importance of primary selected music. For instance, primary music could make humans more powerful and more stimulated during exercise. Moreover, it could effectively decrease the Rating of Perceived Exertion (RPE), which evaluates the degree of effort that a person feels by themselves. Consequently, music could draw attention away from uncomfortable feelings [14].

Nowadays, most playlists are supplied by famous sports brands, which causes unfamiliarity to the users. In addition, the playback mode is typically played by sequence or at random. However, we consider that the previous playback mode is not reasonable during exercise and that the playback sequence should be adjusted depending on the different physical situations of each person. As a result, we propose an algorithm to solve the playback mode during exercise. In order to apply it to individuals, we also consider the biological data and fuzzy algorithm [15]. The fuzzy algorithm is based on the different exercise levels to suggest the canter speed through the biological data of users. We intend to introduce a complete music system consisting of pace match music rhythm, music emotion classification, fuzzy algorithms, and selection music module.

There are many ways to implement emotion classification models. First, simple machine learning models include random forest trees, KNN, and K-means. Although these traditional methods can solve the problem quickly with a small amount of data, they only obtain low accuracy of classification results [16]. Second, RNN [17] has a time series algorithm to solve the emotion classification task. However, the disadvantage of its architecture is prone to generating gradient disappearance and gradient explosion. However, the LSTM and GRU improve time series models to avoid the gradient disappearance and gradient explosion, which need additional parameters to control different gates [17]. To achieve higher accuracy is necessary to overcome the variety of audio in music emotion classification tasks. On the other hand, CNN provides translation-invariant convolution, which can be used to overcome the diversity of audio signals. Thus, we deduce that the model with CNN can improve the classification accuracy in the emotion recognition task based on the characteristics of CNN. From the perspective of practical application, the accelerating technology is more mature than other types, and it brings the possibility of classification with CNN in the audio field.

**Figure 1:**
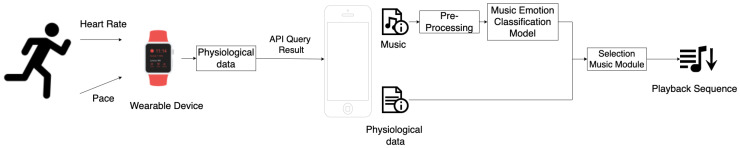
Overview of the proposed playback algorithm. The wearable devices pass the measured signals to smartphone. The smartphone integrates the signals, music elements, and emotion classifier into the algorithm to provide suitable playback mode for individual.

**Figure 2:**
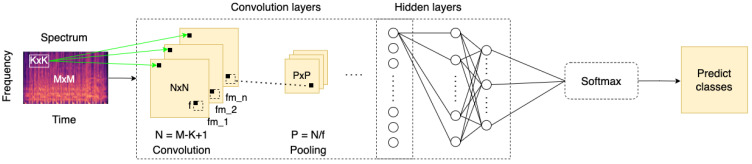
Flowchart of CNN based music emotion classification. Start from original music files, then through convolutional layers and hidden layers, and make predictions with a classifier softmax at the end.

The purpose of this paper is to develop an algorithm for the playback sequence, which adopts CNN-based models for music emotion classification, also considering physiological data adjusting playback sequence immediately, as shown in Figure 1. Figure 2 represents the CNN framework for the music emotion classification. In summary, the key contributions of this paper are:

1. We develop the playlist sequence algorithm concerning physiological data and emotion classification. This innovation can provide the kind of solution that was developed by previous studies.
2. Develop an emotion classification model with multiple kernel sizes and apply it to our proposed playback sequence system.
3. The results from our experiment indicate that the accuracy of the classification strategy is superior to that of other past research.

This paper is structured as follows. Section 2 introduces the existing work on the current research progress of the emotion classification method and summarizes the advantages and disadvantages of the models. Next, Section 3 provides an overview of the model, including the classification and flow of the selected music module. Then, Section 4 conducts the experiment of music datasets on smartphones to evaluate the performance of the models. Finally, Section 5 concludes the research work of this paper and summarizes the overall mentions.

## 2. Related Works

This section will introduce the situation of music intervention during exercise and the traditional deep learning model for music emotion classification based on manual features. Then, we will review classification technologies containing the preprocessing method of audio signals and the progress of deep learning models.

### 2.1. Music Intervention during Exercise

People have considered music to affect the senses since the 18th century, and several researchers have examined the effects of music [18,19]. The primary factors that give rise to the various performances of exercise are the rhythmic, melodic, and harmonic qualities of music. Through synchronizing pace and tempo, people improve their exercise performance. For example, listening to music during exercise can release feelings of discomfort, improving performance and enjoyment [20,21]. The effects of music during physical activity have also been investigated roughly, and there exist several significant points in the literature. Consequently, we will separate the literature into three parts to review the benefits of listening to music during exercise. First, different types of music have different effects on the brain. For example, funk music may make people active, and up-tempo beats make people passionate for working out [22,23,24]. The tone of the music is connected with certain emotions in the music field. For example, the major key typically represents the delighted, and the minor key represents sadness. Second, music can alter the degree of psychomotor arousal (muscular activity associated with mental or movement) and consequently is regarded as either a sedative or a stimulant during physical activities [25,26]. Last, Nikol et al. supposed that humans would simply respond to rhythmical elements in continual extreme activities [27,28]. According to the results, subjects achieve synchronization between movement and tempo; moreover, this can make physical activity become an experience of reducing stress.

The evidence of the existing topic is unanimous and shows that music can affect the ability of attention and a series of emotions, such as increasing work efficiency and motivating rhythmic movements [27,29]. Nikol et al. [27] and Terry et al. [29], who supplied the definition about effects on humans, are unanimous regarding the effect of mild- and low-intensity activities. According to the American College of Sports Medicine(ACSM), exercise intensities are divided by percent of maximum heart rate (MHR), which separates intensities into three stages. For example, low intensity is between 50% and 63% of MHR, moderate intensity is between 64% and 74% of MHR, and high intensity is between 75% and 95% of MHR. Generally, the training zone is between 70% and 85% to keep them healthy. The MHR is calculated as below Equation (1)

(1) MHR=208−0.7∗Age,

where Age is the age of users. However, in high-intensity activities, it is only effective before peripheral fatigue [30]. Consequently, high-intensity exercise typically depends on the Borgs Ratings of Perceived Exertion scale (RPE) [31] to evaluate the degree of fatigue in experiments. The purpose is to prove that music stimuli also exist in high-intensity exercise.

The effects of music on high-intensity exercise are less well documented than low to moderate exercise intensities in the literature. Maddigan et al. [32] designed an experiment about the high intensity of exercise and found that listening to music during activity elicited an increase in exercise duration, breathing frequency, and HR. In light of this, increasing breathing frequency can accelerate oxygen exchange, while HR can balance the requirements of physiology. Although the literature mentioned above has shown that listening to music has many benefits during exercise, the research still has not implemented the playlist order based on the benefits of music yet.

### 2.2. MECS

The music emotion classification systems (MECS) were developed by different researchers in the field of machine learning techniques [33,34]. MECS is used to classify the emotion of music clips, which roughly contains several steps, such as data preprocessing, data collection, feature extraction, and classification. During the data preprocessing stage, the raw data should reform to a standard format for a fair evaluation, such as sample rate (44,100 Hz) and precision (16 bits). Moreover, music emotions are unstable, encompassing different emotions during entire songs. For example, the information of music clips contains abundant features, namely, timbre, energy, rhythm, pitch, and tonality, which stand for the percipient dimensions of songs. In the literature, many researchers mainly adopted the most representative 20–30 s clips of songs for emotion classification [33,34,35]. In order to classify emotions, Russel et al. [36] and Thayer et al. [37] have developed the emotion models, which is used to separate the emotions of songs into different classes.

Music emotion classification through the features of visual representation has been widely explored in a decade, such as Mel-spectrogram, Short-Time Fourier Transform (STFT), and Mel-frequency cepstral coefficients (MFCC). The spectrogram contains a great description of the transient variation of energy distribution on frequency bins. Moreover, the spectrogram is similar to an image and includes enough texture information. There are lots of traditional texture descriptors that have been utilized to describe the content of spectrograms in the computer vision field. These descriptions include local phase quantization, local binary patterns (LBP), Laws texture filter, and Gabor filters. Furthermore, in terms of the classifier, there are some common traditional classification approaches, such as support vector machine (SVM) [38] and Gaussian Mixture Models (GMM) [39], which can train with the above descriptions methods. Although the above traditional classification methods have achieved higher productive results than human-level (60–70%) methods on some music datasets, traditional classification methods still heavily rely on feature engineering [40].

The above method of MECS concerning accuracy is still slightly insufficient. Some of the research continuously tries to improve techniques of classification accuracy. Lee et al. [41] are the pioneers who introduced a deep learning framework to audio classification. Researchers trained a convolutional model that contains two hidden layers and found that the model learns hierarchical features from the preprocessed audio signals. To some degree, Lee et al. inspired future research on deep learning approaches applied to audio classification tasks as feature extractors. Several studies have suggested the benefits of CNN in the audio field; for example, Liu et al. [33] and Bilal et al. [34] used CNN technology on MECS and found that the music emotion classification process was much faster than in previous studies.

**Figure 3:**
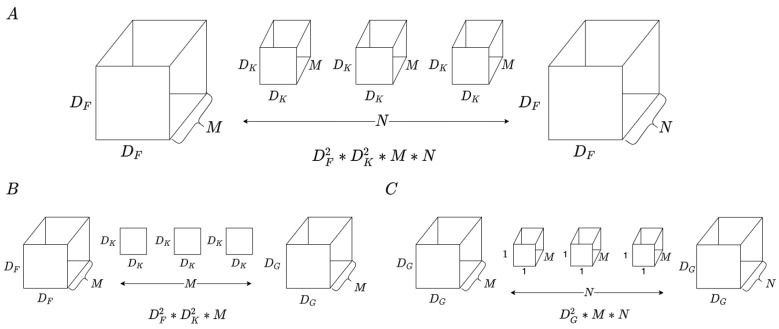
The depthwise separable convolution separated the standard convolution (**A**) into two parts: depthwise convolution in (**B**) and pointwise convolution in (**C**). The 1 × 1 convolution filters are called pointwise convolution in depthwise separable convolution.

There are a lot of deep learning models and machine learning algorithms in the literature; however, they were mostly developed on desktops that had a powerful GPU to process complex computation. Nowadays, the requirement of various applications is increasing with technological advancement. Some practical applications are typically implemented on mobile. By contrast, the mobile is limited by insufficient computation, which leads to the inference time being delayed. As a result, Howard et al. developed the MobileNet [42], which consisted of depthwise convolution and pointwise convolution, as shown in Figure 3B,C. This method reduces the considerable computation compare with the stander convolution, as shown in Figure 3A. The depthwise separable convolution is through expressing convolution as a two-step process.

Inspired by this, we will improve the previous classification models for mobile application. The challenge of this development is to maintain the latency issue while achieving high classification accuracy simultaneously. In this paper, a proposed MECS is developed by considering the following points.

1. The proposed MECS is a classification method that separates the different music types into certain classes by using CNN. In addition, CNN is typically used for feature extraction in deep learning techniques without requiring manual feature extraction [33,34]. Consequently, this method saves a great amount of time for feature extraction. The feature of the spectrogram is learned by different layers of CNN. In order to reduce computation, we adopt the depthwise separable convolution from MobileNet [42] as shown in Figure 3A,B. In order to distinguish the slight differences between each feature, we also adopt the Inception block [43], which contains multiple kernel sizes to extract features. The purpose of this is to consider the slight variation on the spectrogram.
2. The experiments are conducted by using CNN in order to classify the songs in four and eight classes of emotion.
3. 4Q emotion [44] and Soundtrack dataset [45].
4. The training accuracy, training loss, validation accuracy, and validation loss are implemented for all experiments. In addition, the results are compared with the different models on the same dataset to verify the performances.

### 2.3. Selection Music in Exercise Field

Many music selection systems have been developed to aid motivation and performance during physical activities. Im4Sports is one of the first systems to provide customized music based on the music tempo and previously selected songs for users during exercise [12]. Additionally, PersonalSoundtrack [46] and TripleBeat [47] are music players developed explicitly for runners, which sync the user’s running speed (as measured by their heart rate) to the music’s rhythm. With technological progress, wearable devices have gradually replaced complex measurement devices. Additionally, Alaa Khushhal et al. [48] demonstrated the accuracy of wearable devices during exercise. The aforementioned mostly adopted the heart rate to select music with a specific tempo to control the performance of users.

In terms of research advances, music emotion is one of the parameters to affect the performance of users. Recently, Ming-Chuan Chiu et al. [49] considered musical emotions and used machine learning to identify the emotion and select appropriate music based on users’ physiological signals. In this paper, the music playback algorithm took physical activities and musical emotions into account and redesigned the emotion classification method to develop a complete playback algorithm. The selection music module is detailed in the following section.

## 3. Method

In this section, we will first give an overview of the music selection strategy. Then, we will describe the proposed MECS in detail.

### 3.1. Strategy of Selecting Music

**Figure 4:**
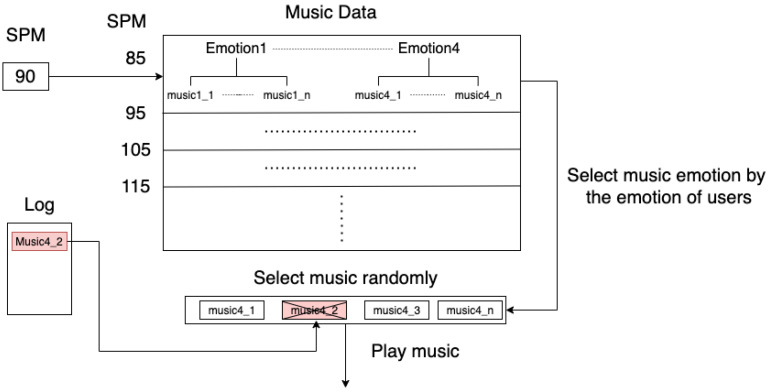
A description of the music selection module.

The Standard Deviation of NN (Normal to Normal) intervals (SDNN) is a significant parameter to represent the emotion of users. However, it is difficult to quantify SDNN during exercise. Contrary to this, measuring HR is more straightforward, so converting HR data to SDNN is required. To implement the strategy of selecting music, we consider user emotion, physiological data, and music emotion as the basis parameters. Music selection approach is divided into the following steps:

1. Detecct User Heart Rate
  We measured the data through the wearable device, which already had signal processing and noise processing in place at the measurement time. Consequently, we used the API to transfer the data directly to the phone for use. In addition, the accuracy of the Apple Watch in the exercise field has been proven by Alaa Khushhal et al. [48].
2. Convert HR Data to R-wave and R-wave interval
  In this study, SDNN is employed to identify the emotion. Directly measuring SDNN is not feasible during exercise because SDNN is analyzed by electrocardiogram, which needs to be measured in a stationary state. Consequently, measuring the HR and converting it to SDNN is feasible. Before converting the HR to SDNN, obtaining the R-wave and R-wave intervals ($RR_{i}$) is required, which represents the elapsed time between two successive R-waves of the QRS-wave signal on the electrocardiogram. To convert HR to $RR_{i}$, we use the following formula [50]:
  (2) $$RR_{i}\approx \frac{60}{HR}.$$
  The deviation of its values from the mean with the root-mean-square (RMS) can determine SDNN. It is obtained by the following formula:
  (3) $$SDNN=\sqrt{\frac{1}{N}\sum_{i=1}^{N}{\left(RR_{i}-\bar{RR_{i}}\right)}^{2}},$$
  where $\bar{RR_{i}}$ represents the mean of the $RR_{i}$.
3. Define emotion of users
  The SDNN values are used to determine the emotion of the user. According to Medicore et al. [51], the feelings of users are roughly separated into four ranges by SDNN, as seen in Table 1. The intervals of SDNN are used to select the music, and the purpose is to maintain the users’ emotions at a certain level.
4. Music Selection Strategy
  The strategy of selecting music is inspired by Chiu, M.C. et al. [49], who proved the relationship between SDNN and music emotion. However, the strategy lacks consideration for the step per minute (SPM) affected by the BPM of music and breathes frequency during exercise. Moreover, the method of music emotion classification is not accurate enough in their experiment. Consequently, in this paper, we improve the method concerning SPM and replace the original method of emotion classification with deep learning. The following example explains the process of selecting music and describing deep learning in the next section.
  We adopt the average BPM of an entire piece of music and group it into different intervals. Each group consists of BPM, which is between n and n + 10. The music selection module will search for each music group that is closest to the SPM of users and then randomly select music in that intervals. In order to avoid repetition situations occurring, the selected music in the latest 10 times will be recorded in the log. More detail is described in Figure 4. For the beginning of Figure 4, the input of selecting music module is 90, and the emotion1, emotion2, emotion3, and emotion4 are the group of music data. In this example, music1_1 to music1_n represent music in the emotion1, music2_1 to music2_n represent music in the emotion2, and so on. The reason for selecting music is because it is located in a music group with 85–95 and corresponds to the emotions of users. We assume that the corresponding group of music is emotion4. Furthermore, music4_2 is not selected because it has been recorded in the log, indicating that it has already been played. As a result, the system will select the other music in the emotion4 and automatically exclude music4_2 simultaneously.
5. Music Playing Strategy
  The strategy of playing music is chosen by the previously mentioned method. In addition, the purpose is to lower energy consumption as much as possible. As we know, the inference process is the most energy-consuming. Consequently, we reduce the usage of the emotion classification model by the following Algorithm 1. Consequently, we design the algorithm to reduce energy consumption. This main process includes reducing the classification usage, reserving the 10 songs, which correspond to the emotion of users, not classification all of the music. When the step frequency changes, the system will select the music in the piece of the group based on current SPM, also considering the emotion of users to give corresponding emotion. This function allows the users to always listen to music at an appropriate pace for their own step frequency and provides a proper emotion to keep users steady. Furthermore, all operations are automatic in the process. Subsequently, we elaborate the parameters as below. First, we denote *S* as SPM and denote $\bar{S}$ average SPM of the last 10 times. Second, $B_{now}$ represents the current BPM of the playing music, and *D* is denoted as the number of times of deviation between BPM and SPM. Last, the count represents a counter, and uBound represents the upper limit of times, which is out of the range deviation of *S* and $B_{now}$. The strategy of playing music is presented in the following manner:
  In summary, this algorithm considers the physiological information and adopts the proposed music emotion classification model in the overall process. As we know, the process of inference has the most consumption of power. In order to ensure the application can operate for a long time on mobiles, we developed music played strategy to reduce the usage of MECS. In the following section, we will propose the MECS with low parameters, FLOPs (floating-point operations per second), and high accuracy, to achieve fast inference time on the mobile.

**Algorithm 1:** The music playing strategy with SPM and emotion
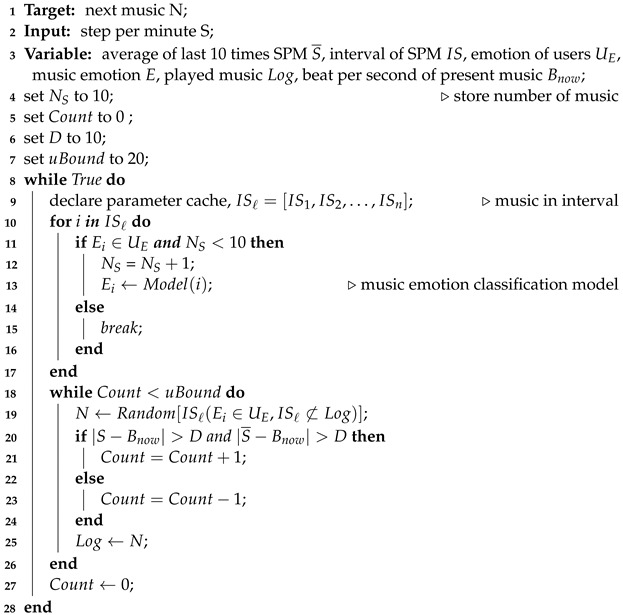


### 3.2. Music Emotion Classification System

In this section, three parts were divided to elaborate on the process of music emotion classification. To begin with, we introduce the architecture of the residual–inception block in detail, then explain the benefit of the separable depthwise convolution on the blocks. Finally, we illustrate the overall architecture and indicate the configuration of the model.

#### 3.2.1. Residual Connectivity with Inception Block

**Figure 5:**
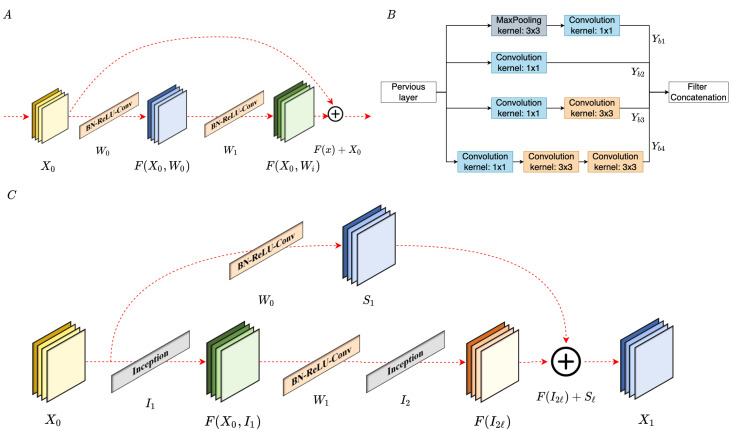
(**A**) Residual connectivity. (**B**) Inception block. (**C**) Residual-Inception block. (**A**,**B**) have two identical points: one thing is that both use the particular connection method to reserve the original feature as much as possible; the other thing is that both networks use 1 × 1 convolution as a bottleneck layer to reduce the computation cost. The difference between (**A**,**B**) is the method of output tensor difference; (**A**) adopt the add layer, while (**B**) adopt the concatenate.

Residual connectivity [52], Inception [43], and residual–inception blocks are depicted in Figure 5. The methods mentioned above improved the model classification accuracy through different operations, which are implemented through the connection method and multi-kernel size filters, as represented in Figure 5A,B, respectively. The proposed model adopts the inception block with residual connectivity to extract the subtle changes and simultaneously keep the gradient stability, as shown in Figure 5C.

Residual connectivity provides the advantage of shortening connections, allowing feature maps to be learned by calculating the difference between input and output features. The residual connectivity block is depicted in Figure 5A. The layer obtains the residual feature maps of the previous layers and then adds the residual feature and original feature maps. The $\ell_{th}$ expresses the number of block depths, and $X_{\ell }$ is used as an input to the residual blocks. Next, the feature extraction is denoted as $W_{i}$. $F(X_{\ell },W_{i})$ denoted as the features of the preceding layers. Additionally, $X_{\ell }$ and $W_{i}$ are the functions of x, so we denoted them briefly as F(x).

$W_{i}$ is the function of three successive operations, consisting of rectified linear unit (ReLU), batch normalization (BN), and 3 × 3 convolution (Conv). However, the significant characteristics of individual sound types are distinguished by their different frequency bands and temporal intervals. Music is regarded as a kind of audio with more features than audio; besides, music includes many specific elements, such as timbre, rhythm, and pitch. In addition, music has different features regarding frequency and time selection in various genres. As a result, the spectrum of music is very sensitive to distinct genres. The benefit of inception is that it can accept multi-level feature maps using a mix of various kernel sizes. The performance network is improved by combining multi-level feature maps from various filters. Additionally, before the inception blocks were proposed, all architectures conducted convolution on the spatial and channel-wise domains. The inception blocks could roughly separate the steps into two parts. First, by performing cross-channel correlations using the 1 × 1 convolution. Second, through cross-channel correlations and cross-spatially through the 3 × 3 and 5 × 5 filters, as shown in Figure 5B.

In the proposed structure, residual connectivity is utilized to connect two inception blocks and shortcut layers, which are represented in Figure 5C. Based on previous research, each residual block can keep the original information, simultaneously learning the residual features. We denote $X_{L}$ as the output of shallow layers and $S_{L}$ as the output of shortcut layers. Both pass through the add layer, then the combination features are the input of the residual blocks. Among them, the *L* is the number of residual blocks. The proposed model layer is similar to inception, which carries out a non-linear transformation $I_{\ell }\left(.\right)$, where *ℓ* is the index of the layer. $I_{\ell }\left(.\right)$ is a composite operation function, including the BN, ReLU, Conv, or Pool. In the music emotion classification task, we fixed *L* = 2. Thus, the input of $\ell_{th}$ block, ℓ=1,⋯,L, while output can be represented as $x_{\ell }$:

(4) $$x_{\ell }=add\left(S_{\ell },I_{2\ell }\left(\left[Y_{b1},Y_{b2},Y_{b3},Y_{b4}\right]\right)\right),$$

where $S_{\ell }$ is the output of shortcut; $Y_{b1}$, $Y_{b2}$, $Y_{b3}$, and $Y_{b4}$ are outputs of the Inception block; and [$Y_{b1}$, $Y_{b2}$, $Y_{b3}$, and $Y_{b4}$] are the filter concatenation. Feature maps are concatenated through the channel dimension at each inception block.

#### 3.2.2. Depthwise-Separable Convolution

**Figure 6:**
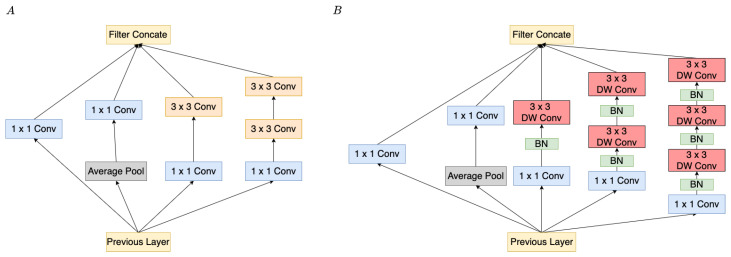
(**A**) shows the standard inception block. (**B**) represents the modified inception block. The difference between (**A**,**B**) is that (**B**) replaces the standard convolution with depthwise separable convolution. Moreover, the BN layer is introduced before the 3 × 3 convolution.

Inspired by MobileNet [42], the standard convolution layer on the residual–inception block is replaced with a depthwise-separable convolution, which can effectively reduce the computation. The structure of depthwise-separable convolution layers contains a depthwise convolution layer and a pointwise convolution (or called 1 × 1 convolution). Compared to the standard convolution, the depthwise-separable convolution can effectively reduce the overall computations by separating the convolution into two parts. We present the computation of a standard convolutional layer, and a pointwise convolutional layer; a depthwise convolutional layer is defined as (5)–(7), respectively. The variable names are described as follows: $D_{F}$ represents the input feature size, kernel size is denoted as $D_{K}$, the number of channels is denoted as *M*, and the number of output channel is denoted as *N*. The reduction of overall computation is simplified as (8)

(5) $$D_{F}^{2}*D_{K}^{2}*M*N,$$

(6) $$D_{G}^{2}*M*N,$$

(7) $$D_{F}^{2}*D_{K}^{2}*M,$$

(8) $$\frac{D_{F}^{2}*D_{K}^{2}*M+D_{F}^{2}*M*N}{D_{F}^{2}*D_{K}^{2}*M*N}=\frac{1}{N}+\frac{1}{D_{K}^{2}},$$

where the overall computation cost depends on the number of parameters, such as input feature map $D_{F}*D_{F}$ (assume the length and width of feature maps are identical), input channel *M*, kernel size $D_{K}$, and the number of output channels *N*. More specifically, the depthwise-separable convolution layer can reduce approximately 80% of the computation on 3 × 3 convolution layers, compared with the standard convolution. Although the accuracy will slightly decrease while reducing the amount of calculation, we consider these losses acceptable. Accordingly, the proposed structure differs from the standard inception block. In addition, we employ an extra BN layer before each convolution, which significantly enhances the network learning ability even when training on a small-scale dataset. In addition, we adopt stack three layers 3 × 3 convolution to extract much more different features, as shown in Figure 6.

#### 3.2.3. Network Architecture

**Figure 7:**
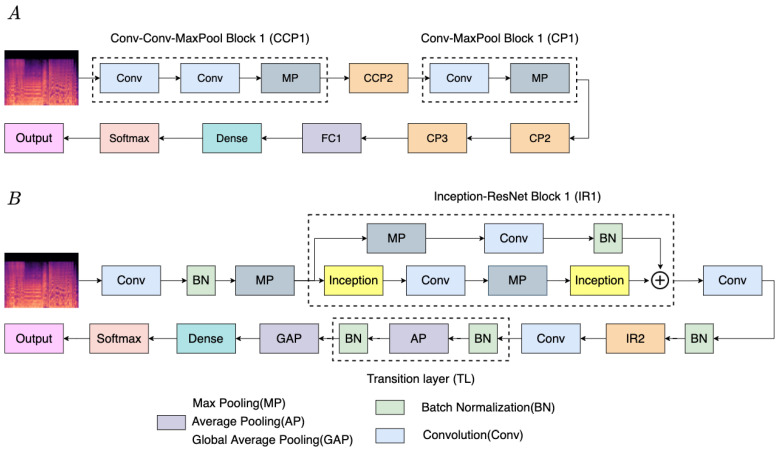
(**A**) the music emotion classification based on VGG Net [54]. (**B**) The overall propose architecture.

The proposed model consists of 10 layers when only calculating the layers with parameters. More specifically, the proposed model mainly comprises convolution layers and residual–inception blocks, which all implement Conv, Softmax, BN, and max-pooling operations in each layer. Inspired by [53], this study adopts the BN operation between each convolution and the ReLU, whose purpose is to calculate the root mean square of features to perform normalization for each batch. Our emotion classification architecture is developed based on Sarkar et al. [54], which is built around VGG (visual geometry group) Net. Moreover, it has a higher classification accuracy than the other researchers. We visualize the network architecture as depicted in Figure 7A. Sarkar et al. [54] consider fewer layers than standard VGG Net, and their method alleviates the problem of overfitting on small-scale training datasets. They stack two convolution layers, followed by a max-pooling layer as a module (Conv-Conv-Pooling (CCP)). Moreover, their network structure is stacked by three or four modules.

The proposed model is presented by the following steps: to start with, the 3 × 3 convolution is used as a basic feature extractor, adding BN and a ReLU after each convolution layer. After that, the kernel size of the max-pooling layer is 1 × 5, which is used to keep the most significant features of the feature maps. Next, we replace their CCP module with the residual–inception block and design the network structure by stacking it twice. In addition, considering that the characteristic of inception is concatenation, each branch needs to set the same number of filters. In this study, all of the convolution layers use 32 filters in the inception blocks. Then, between two residual–inception blocks, we adopt the 1 × 1 convolution to reduce the dimension of channels. Finally, the transition layer is under consideration and is typically used to reduce the complexity of feature maps. The transition layer makes the channel of feature maps become half of the original by average pooling, which is connected after the residual–inception blocks in our structure.

The traditional CNN almost generates the network parameters of 80% on the last few fully connected layers. Accordingly, we replace the fully connected layer with global average pooling (GAP) [55]. The considerable parameters are not easy to use to calculate the fit regularizer, resulting in the weak generalization ability of the model, further bringing out the overfitting. Compared to the fully connected layer, the strength of GAP lies outside of the extra parameters, further avoiding the problem of overfitting. It operates through the average of all feature maps on spatial information. Thus, it is more robust to spatial translations of the feature maps. In addition, GAP is a regularizer, which forces the final output feature maps to be directly mapped to the categories confidence maps. Finally, it puts the feature maps that have averaged into softmax to obtain probability classification, and the cross-entropy is the selected loss function, which provides a smooth gradient to make computation much simpler.

The proposed architecture considers computational efficiency and practicality. Accordingly, the model has the strength of a small model size and a certain accuracy level. The configuration of architecture is described in Table 2. As illustrated, the 128 × 1100 × 1 features are considered as the input of the model. In all convolution layers, we pad zeros to each convolution to keep the size fixed. As shown in Table 2, the trained model has tiny size, only 2.4 MB. It contains the model and weight information applicable to personal devices, even those limited by computation resources.

## 4. Experiment Results

The structure of the section is divided into several subheadings to describe the experiment results in detail. First of all, the dataset description is provided. Next, we illustrate the data preprocessing in our experiments. After that, we compare the proposed model with other research models and analyze the performance. Lastly, we implement the proposed model on the mobile and present the energy consumption of the overall automatic music selection system.

### 4.1. Dataset

This study utilizes the 4Q emotion [44] and the Soundtrack [45] datasets. The sample rate of audio clips is 44.1 kHz in the above datasets, which are described below in detail.

**Bi-modal**. This dataset consists of 162 songs. Each song clip is of 30 s duration. The emotion category is also annotated into four A-V quadrants by Russell’s model. In this dataset, each emotion category has a different number of music clips, Q1: 52 clips; Q2: 45 clips; Q3: 31 clips, and Q4: 34 clips. In addition, these 162 songs are annotated with four quadrants. The quadrants are Q1 (A+V+), Q2 (A+V−), Q3 (A−V+), and Q4 (A−V−), which correspond to happy, anger, sad, and tender, respectively.

**4Q emotion**. This dataset [44] consists of 900 songs, and the duration of each clip is 30 s. In addition, these 900 songs are annotated with four quadrants. The quadrants are Q1 (A+V+), Q2 (A+V−), Q3 (A−V+), and Q4 (A−V−), which correspond to happy, anger, sad, and tender, respectively.

**Soundtracks**. The dataset [45] consist of 360 audio samples, which are taken from the background tracks of films with a length of approximately 30 s. Each clip is labeled with a distinct emotion category, such as wrath, sadness, happiness, fear, surprise, valance, energy, and tenderness. A clip may have several tags that have different levels of confidence. In the experiment, in order to clarify the category of dataset, we combine the high and low classes into the same classes. For example, low energy and high energy are in the same emotion category. Consequently, there are nine classes in our experiments.

### 4.2. Data Preprocessing

Han, Yoonchang et al. [56] used Mel-spectrogram with 128 Mel filters for the time-frequency representation of the audio. They claimed that the 128 Mel filters contain sufficient spectral characteristics while significantly reducing the feature dimension. Mel-spectrogram is used as the input to the proposed network in this study, which is achieved by adding a logarithmic scale to the frequency axis of the STFT function. To prove if the 128 Mel filters achieve the best accuracy in our task, we implemented the experiment with four different Mel filters. Thus, we set the 30, 60, 90, and 128 Mel filters in the experiments. The parameter determined the shape of the feature vector before the feature extraction process. Specifically, we extracted the Mel-spectrogram with 30, 60, 90, and 128 Mel filters (bins) using the librosa tool [57], setting the hop length as 1024, frame size as 2048, window function as the Hamming window, and sample rate as 44,100 Hz. When assigning the above parameters, we used the suitable vector of Mel-spectrogram as the input feature.

### 4.3. Data Augmentation

Data augmentation is a technique for avoiding model overfitting by augmenting the number of data used in model training. There are three different augmentation methods based on the particular qualities of music. The first is time overlapping, which is a useful technique in picture processing. Window shifting of audio signals generates extra data by adjusting the overlap to 50% to enhance the valid data size. Second, background noise was added to the signal, which added a signal-to-noise ratio of 10 dB to implement the augmentation of the data. The last was pitch shifting, which shifts the pitch of audio clips. We lowered the pitch of a waveform by a semitone. Under this process, the slight perturbations increased sample diversity but not impact the original music expression. Consequently, when we finished the data augmentation process, three times the original data remained. The results of augmented data are shown in Table 3 and Table 4.

### 4.4. Training and Other Details

The preprocessing method converts the raw data into Mel-spectrogram, which is described in Section 4.2. The model utilizes the Mel-spectrogram with 128 × 1100 as input, and it is trained by using the ADAM [58] optimizer to reduce categorical cross-entropy between predicted and actual labels. Each dataset was trained with 100 epochs and a batch size of 32. The learning rate starts with 0.01 and reduces it by a factor of 0.5 automatically after five epochs if the loss does not decrease. In order to validate the robustness of the model, we adopted k-fold cross-validation to evaluate the performance of the proposed model on the 4Q emotion and the Soundtrack datasets. In all datasets, we separated the data into training, testing, and validation at 80%, 10%, and 10%, respectively. The experiment was developed in Python, and the model was trained by using the Keras, Tensorflow, and Librosa toolkits on NVIDIA RTX-3090 GPU with 24 GB RAM. We measured the inference time of all the models on the iOS mobile device, iPhone 8 Plus, converting the trained model to ML model (.mlmodel).

### 4.5. Results on the Bi Modal Dataset

This following section describes the performance of the proposed model and classification results. First, comprehensive comparisons with other models, including the number of parameters, accuracy, inference time, and total FLOPs, are provided on Bi-modal dataset and 4Q emotion dataset. The inference time is estimated on iOS mobile devices in this experiment. Afterward, to emphasize the robustness of the proposed model and three available models, we examined the soundtrack dataset consisting of nine classes for testing emotion classification. This study also provides various comparisons with different models to estimate the performance by the following indicators, including total FLOPs, overall parameters, the accuracy, and the result of cross-validation.

Table 5 exhibits the performance of different technologies on the Bi- modal dataset. It can be observed that the proposed method attained an accuracy of 84.91%, which is better than Sarkar et al. (81.03%) [54], VGGNet (63.79%), Inception v3 (64.15%), and ResNet (77.36%). The results indicate that the proposed model has great advantages of accuracy. Moreover, we implement the architecture proposed by Sarkar et al. and validate the result on the Bi-modal dataset. In the following section, we use the same configuration with Sarkar et al. proposed model to test different datasets and compare them with ours.

### 4.6. Results on the 4Q Emotion Dataset

Table 6 exhibits the performance of various designs on the 4Q emotion dataset. It can be seen that the proposed method achieved an accuracy of 92.07% on the 4Q emotion dataset. The accuracy is 20.32% and 15.23% higher than those of the SVM with ReliefF (baseline) [44] and VGG-16, respectively. In comparison with Chaudhary [59], the accuracy of the proposed model improved by 3.65% and FLOPs of the model were reduced by 68%. In addition, comparing our model with CCP [54], the accuracy rose by 5.56%, and the training parameters and FLOPs by 89.8% and 89.3%, respectively. The results indicated that the proposed model has significant advantages of computation and accuracy. Furthermore, our approach outperforms VGGNet, Inception, and ResNet in terms of classification accuracy by 15.23%, 9.6%, and 7.21%, respectively. The experimental results indicate that our residual–inception block-based model is effective.

**Figure 8:**
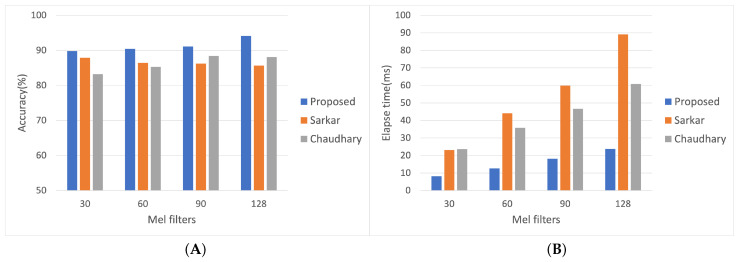
(**A**) represents the result of classification accuracy comparison of the proposed system with the other architecture. The y-axis accuracy (%) means the number of correct classifications/the number of all data. (**B**) shows the result of elapsed time in classifying audio file comparison the proposed system with two different models.

The proposed model has the most remarkable accuracy and the fewest parameters, with lower FLOPs and better accuracy than the others. Classification accuracy comparison between the proposed system and two other architectures is presented in Figure 8 for emotion classification. It can be observed that the proposed model has higher accuracy than other models at the following four different frame length experiments. The Mel filters of 128 produce the highest classification accuracy compared to the other experiments; the accuracy of different Mel filter sizes with 30, 60, 90, and 128 are 89.76%, 90.42%, 91.11%, and 92.07%, respectively.

### 4.7. Results on Soundtrack Dataset

We compare the proposed model with some recent models, including three different deep learning models and two traditional classification methods, as shown in Table 7. Sarri et al. [60] classified the features with SVM and k-NN on the Soundtrack dataset, fetching an accuracy of 54%. The result is obviously insufficient, so the neural network as the classifier gradually replaces SVM and k-NN. The development of the VGG network replaces the old technique of extracting features, resulting in the accuracy substantially rising by 12%. Moreover, the RNN-based architecture was developed, which can describe dynamic time behavior and possesses the ability to extract the slight changes on the spectrogram. The MCCLSTM [61] consists of long short-term memory (LSTM) and CNN, and the result demonstrates that it achieves an accuracy of 74.35 %. However, when the accuracy needs to be further improved, the number of parameters needs to be kept low at the same time. The LSTM-based architecture is not easy to implement in low parameters because the LSTM unit parameter requires four times more than the CNN-based architecture. As a result, the CNN-based architecture represents a significant direction of development. Sarkar et al. committed to improving the VGG network and developing the CCP module to replace the original VGG network. There are some benefits of the CCP module, described as follows. First, CCP alleviates the issue of overfitting on small training datasets. Second, the CCP module achieved higher accuracy than the VGG network, which achieved an accuracy of 82.54%. Finally, Chaudhary et al. utilized a different architecture of convolutional layer kernel, fetching an accuracy of 83.98%. In addition, Chaudhary et al. developed architecture through stacking various kernel size convolutions and achieving higher accuracy and lower FLOPs. However, the accuracy is still lower than the proposed models, and FLOPs are higher than ours. According to the experiment results, the accuracy of the proposed model outperforms all the compared models, whether on the 4Q emotion and Soundtrack dataset.

**Figure 9:**
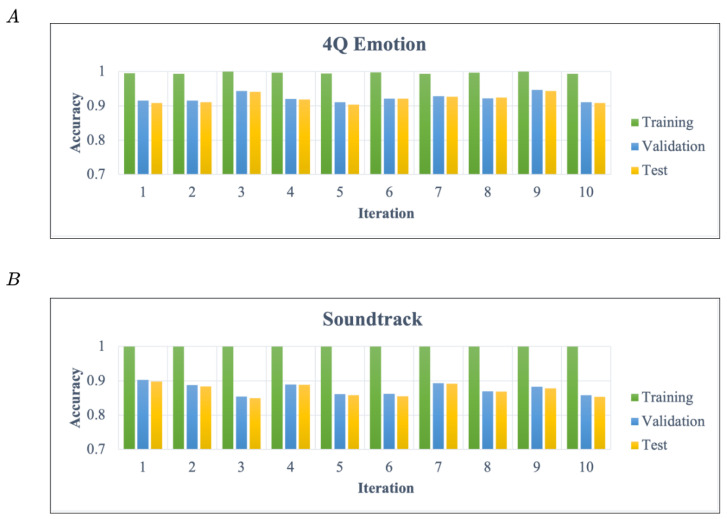
(**A**) represents the result of cross validation on the 4Q emotion dataset, and (**B**) shows the result of cross validation on Soundtrack. (**A**,**B**) are separated the individual dataset into ten times iteration to validate the model performance. The average of (**A**) (4 classes) is 92.07%, and the average of (**B**) (9 classes) is 87.24%.

To prove whether the model generalizes well to new data, we performed a 10-fold cross-validation on each dataset. Figure 9 illustrates the training, testing, and validating accuracy in each iteration. The training, testing, and validation data are 80%, 10%, and 10%, respectively. The experimental results show that the classification results of the different datasets and the new data achieve a competitive level to represent the proposed model possessing generalization ability.

### 4.8. Runtime of the Developed Application

The strategy of playing music is chosen by the previously mentioned method. In addition, the purpose is to lower energy consumption as much as possible. As we know, the inference process is the most energy-consuming. Therefore, we reduce the usage of the emotion classification model by developing the following algorithm to reduce energy consumption. As a result, we design the algorithm to reduce energy consumption. This main process includes reducing the classification usage and reserving the 10 songs that correspond to the emotion of users, and does not classify all of the music to achieve the lower power consumption. The Table 8 depicts the energy usage of the proposed system during the process.

## 5. Conclusions

This paper presented a specific network for detecting music emotions while running the music selection system. The proposed model intends to use the low-level information in log-scaled Mel-spectrograms to make a classification choice. The proposed method is proven competitive with existing deep learning architectures on the 4Q emotion and the Soundtrack datasets. Furthermore, four different numbers of Mel filters are used to generate the input spectrograms. We discovered that the increasing number of Mel filters results in higher classification accuracy as well. Thus, we can deduce that the more Mel filters we set, the more features we obtained. Specifically, the proposed model achieves 84.91%, 92.07%, and 87.24% on Bi-modal, 4Q emotion, and Soundtrack datasets, respectively, higher than other emotion classification models, and the inference time is lower as well. The proposed emotion classifier will be used in the field of music therapy. In addition, this paper designed a selection module based on a set of physiological data of users and music emotional variables for when the user is running. Furthermore, we lessened the energy consumption by reducing the usage of the emotion classification model to ensure that it can execute for a long time on mobiles.

In summary, this study developed an entire system for joggers, solving the problem of playback sequence, which has not considered the present physiological and music emotion during exercise in the previous studies. The entire playback sequence consists of the classifier and music selection module developed based on previous research on music interventions. The classifier can be used as part of the music playback sequence. The system will change music sequence immediately to make users exercise more efficiently, according to the present situation of physiological data.

## Figures and Tables

**Table 1:** Emotion interval.

Emotion	Sleep	Boredom	Anxious	Panic
SDNN range	<20	20~34	35~49	>50

**Table 2:** The configuration of proposed model. The dimension of output shape represent in frame length, Mel features, and channel).

Layer	Filter Size/Stride (Number of Filters)	Out Shape	Params
Input	**-**	(128, 1100, 1)	**-**
Conv1	3 × 3/1 (32)	(128, 1100, 32)	448
MaxPool1	1 × 5/None	(128, 220, 32)	0
Inception1(front)	[3 × 3/1 (32)avergepool] * 1,		
	[1 × 1/1 (32)conv] * 5,	(128, 220, 160)	14,272
	[3 × 3/1 (32)conv] * 6		
MaxPool2	2 × 2/None	(64, 110, 160)	0
Inception1(behind)	[3 × 3/1 (32)avergepool] * 1,		
	[1 × 1/1 (32)conv] * 5,	(64, 110, 160)	35,392
	[3 × 3/1 (32)conv] * 6		
MaxPool(shorcut1)	2 × 2/None	(64, 110, 32)	0
Conv(shorcut1)	1 × 1/1 (160)	(64, 110, 160)	5920
Add1	-	(64, 110, 160)	0
Conv2	1 × 1/1 (32)	(64, 110, 32)	5312
Inception2(front)	[3 × 3/1 (32)avergepool] * 1,		
	[1 × 1/1 (32)conv] * 5,	(64, 110, 160)	14,272
	[3 × 3/1 (32)conv] * 6		
MaxPool3	2 × 2/None	(32, 55, 160)	0
Inception2(behind)	[3 × 3/1 (32)avergepool] * 1,		
	[1 × 1/1 (32)conv] * 5,	(32, 55, 160)	35,392
	[3 × 3/1 (32)conv] * 6		
MaxPool(shorcut2)	2 × 2/None	(32, 55, 160)	0
Conv(shorcut2)	1 × 1/1 (160)	(32, 55, 160)	5920
Add2	-	(32, 55, 160)	0
Conv3	1 × 1/1 (32)	(32, 55, 32)	5280
AveragePool	2 × 2/2 None	(16, 27, 32)	0
GlobalAveragePool	**-**	(32)	0
Dense	Number of Classes	(Number of Classes)	132
		**Total Params**	**122,340**

**Table 3:** The amount of data on Bi-Modal and 4Q emotion dataset.

Dataset	Q1	Q2	Q3	Q4	Total
Bi-Modal	156	135	93	102	486
4Q emotion	675	675	675	675	2700

**Table 4:** The amount of data on Soundtrack dataset.

Dataset	Anger	Energy	Fear	Happy	Sad	Surprise	Tender	Tension	Valance	Total
Soundtrack	116	224	116	124	112	112	112	236	232	1279

**Table 5:** On Bi-Modal datasets, the result present as bold.

Method	Accuracy	Param	FLOPs
VGG-16	63.79%	14,992,068	85,681,045,528
Inception-v3	64.15%	21,810,404	15,803,943,448
ResNet-18	77.36%	13,994,372	11,539,579,288
Sarkar et al.	81.03%	1,203,140	17,710,549,784
**Ours**	**84.91%**	**122,340**	**1,878,742,552**

**Table 6:** On 4Q emotion datasets, the result present as bold.

Method	Accuracy	Param	FLOPs
SVM + ReliefF	71.75%	-	-
VGG-16	76.84%	14,992,068	85,681,045,528
Inception-v3	82.47%	21,810,404	15,803,943,448
ResNet-18	84.86%	13,994,372	11,539,579,288
Sarkar et al.	86.42%	1,203,140	17,710,549,784
Chaudhary et al.	88.47%	375,012	5,922,046,296
**Ours**	**92.07%**	**122,340**	**1,878,742,552**

**Table 7:** On Soundtrack datasets, the result present as bold.

Method	Accuracy	Param	FLOPs
SVM BE of	54.63%	-	-
k-NN BE of	56.45%	-	-
VGG-16	68.12%	14,992,068	85,681,045,528
MCCLSTM	74.35%	2,487,978	1,230,683,852
Sarkar et al.	82.54%	1,203,140	17,710,549,784
Chaudhary et al.	83.98%	375,012	5,922,046,296
**Ours**	**87.24%**	**122,340**	**1,878,742,552**

**Table 8:** Resources consumption of the developed system in the situation of jogging.

Index	CPU	RAM	Max. CPU Usage	Max. Memory Consumption
Phone A	A10 Fusion	2 GB	34.6%	228.1 MB
	Quad-core 2.34 GHz			
Phone B	A11 Bionic	4 GB	18.1%	247.5 MB
	Hexa-core 2.39 GHz			
Phone C	A12Z Bionic	6 GB	13.7%	237.7 MB
	Hexa-core 2.49 GHz			
Phone D	A14 Bionic	6 GB	14.7%	178.4 MB
	Hexa-core 2.99 GHz			

## Data Availability

Not applicable.
